# ICTs and interventions in telerehabilitation and their effects on stroke recovery

**DOI:** 10.3389/fneur.2023.1234003

**Published:** 2023-08-14

**Authors:** Yanghui Xing, Jianxin Xiao, Buhui Zeng, Qiang Wang

**Affiliations:** ^1^Department of Biomedical Engineering, Shantou University, Shantou, China; ^2^National Research Center for Rehabilitation Technical Aids, Ministry of Civil Affairs, Beijing, China

**Keywords:** telerehabilitation, telehealth, stroke, information and communication technologies, rehabilitation

## Abstract

Telerehabilitation (TR) is a new model to provide rehabilitation services to stroke survivors. It is a promising approach to deliver mainstream interventions for movement, cognitive, speech and language, and other disorders. TR has two major components: information and communication technologies (ICTs) and stroke interventions. ICTs provide a platform on which interventions are delivered and subsequently result in stroke recovery. In this mini-review, we went over features of ICTs that facilitate TR, as well as stroke interventions that can be delivered via TR platforms. Then, we reviewed the effects of TR on various stroke disorders. In most studies, TR is a feasible and effective solution in delivering interventions to patients. It is not inferior to usual care and in-clinic therapy with matching dose and intensity. With new technologies, TR may result in better outcomes than usual care for some disorders. One the other hand, TR also have many limitations that could lead to worse outcomes than traditional rehabilitation. In the end, we discussed major concerns and possible solutions related to TR, and also discussed potential directions for TR development.

## 1. Introduction

Stroke is a leading cause of death and disability globally (1). The occurrence of stroke is increasing rapidly in terms of absolute numbers due to population aging (2). Worldwide in 2019, there are 143 million stroke survivors suffering from various symptoms such as hemiplegia, aphasia and depression, which greatly impair their independency and cause tremendous burden to patients, their family and the society (3, 4). Extensive studies demonstrated that proper rehabilitation programs can ease stroke symptoms, reduce long-term disability, and improve quality of life (5, 6). Patients should start rehabilitation as early as possible in order to prevent chronic damage to the brain (7), and continue to do so even after recovery is slower than before (8). Traditionally, rehabilitation services are provided by healthcare professionals in clinic settings. But this is difficult for patients living in remote areas, especially in low- and middle-income countries. They have no access to rehabilitation services or they have to take extra time and efforts to travel a long distance. In this situation, telerehabilitation (TR) can offer an alternative way to deliver services (9).

By using information and communication technologies (ICTs), TR is able to minimize the barrier of distance between patients and rehabilitation providers (10). The role of ICTs is to ensure traditional in-clinic rehabilitation services delivered remotely to patients as effectively as possible. TR is not a new subspeciality (9); instead, it covers all aspects of rehabilitation, including “evaluation, assessment, monitoring, prevention, intervention, supervision, education, consultation, and coaching (11).” There are a number of advantages to use TR for stroke patients. TR can save time and money, make the access to healthcare professionals easier, and provide extra training opportunities for interventions requiring higher dose. It also helped to decrease infection rates of certain diseases, and may provide emotional support to patients for being at home (12, 13).

In this mini-review, we will focus on two major components of TR: ICTs and stroke interventions, as well as outcomes of TR for various stroke conditions. ICTs serve as platforms on which interventions are delivered. Both of them are the keys to feasibility, effectiveness and safety of TR as well as to patients’ satisfaction and adherence. Previous reviews regarding stroke and TR are mainly focused on one aspect, such as upper limb rehabilitation or application of virtual reality (14, 15). So, we think it is necessary to provide an overall picture in order to summarize key factors in this topic. In the end, we also discussed existing issues and potential future development.

## 2. Information and communication technologies in telerehabilitation

ICTs are the foundation of TR, allowing stroke survivors to achieve optimal recovery outcomes by utilizing home-based therapies (16). There are a number of ICTs available, including text, audio, visual, mobile-based, computer-based, web-based, sensors and wireless devices (17). The major purpose of ICTs is to provide a platform for patients to receive rehabilitation services as if in clinical settings. The platform should be safe, user friendly and feasible to apply stroke interventions to all users with high tolerance for error (18). When possible, the platform should be easily modified to deliver personalized service. The considerations of building up a TR platform involve a variety of factors, such as effectiveness of intervention, customer support, cost, accessibility, usability and acceptability (19). For example, low-cost platform may be not effective enough but can be afforded by most people; while high-cost platform usually is more complicated with a higher learning curve resulting in less use.

Telephone is one of the earliest TR methods and still frequently used today. In a recent study by Cha et al. (20), nurse-initiated phone call interventions are able to increase physical activities of subacute patients after hospital discharge. Calls to discuss patients’ conditions may also increase their adherence to therapy and satisfaction with it (21). It is also used in goal setting programs for self-management of daily activities and stroke knowledge education (22). These results suggest that low-cost solutions, such as telephone and text, are still viable in plenty of situations.

Videoconferencing is an upgrade option of telephone service, providing both audio and visual communication between patients and healthcare professionals. Videoconferencing can be mobile-based or computer-based to support face-to-face information exchange. Li et al. (23). investigated feasibility, validity, and reliability of using videoconferencing for functional assessments of stroke patients after hospital discharge, and telephone service and home visit were used as controls. The functional status of patients was measured at the end of 2 weeks and 3 months. The authors found that patients offered videoconferencing and home visit have similar scores in functional status. Videoconferencing has higher validity and reliability than telephone based on measures from this study. Patients in videoconferencing group also showed high satisfaction and confidence. The results suggest that videoconferencing is a better solution than telephone.

**Figure 1:**
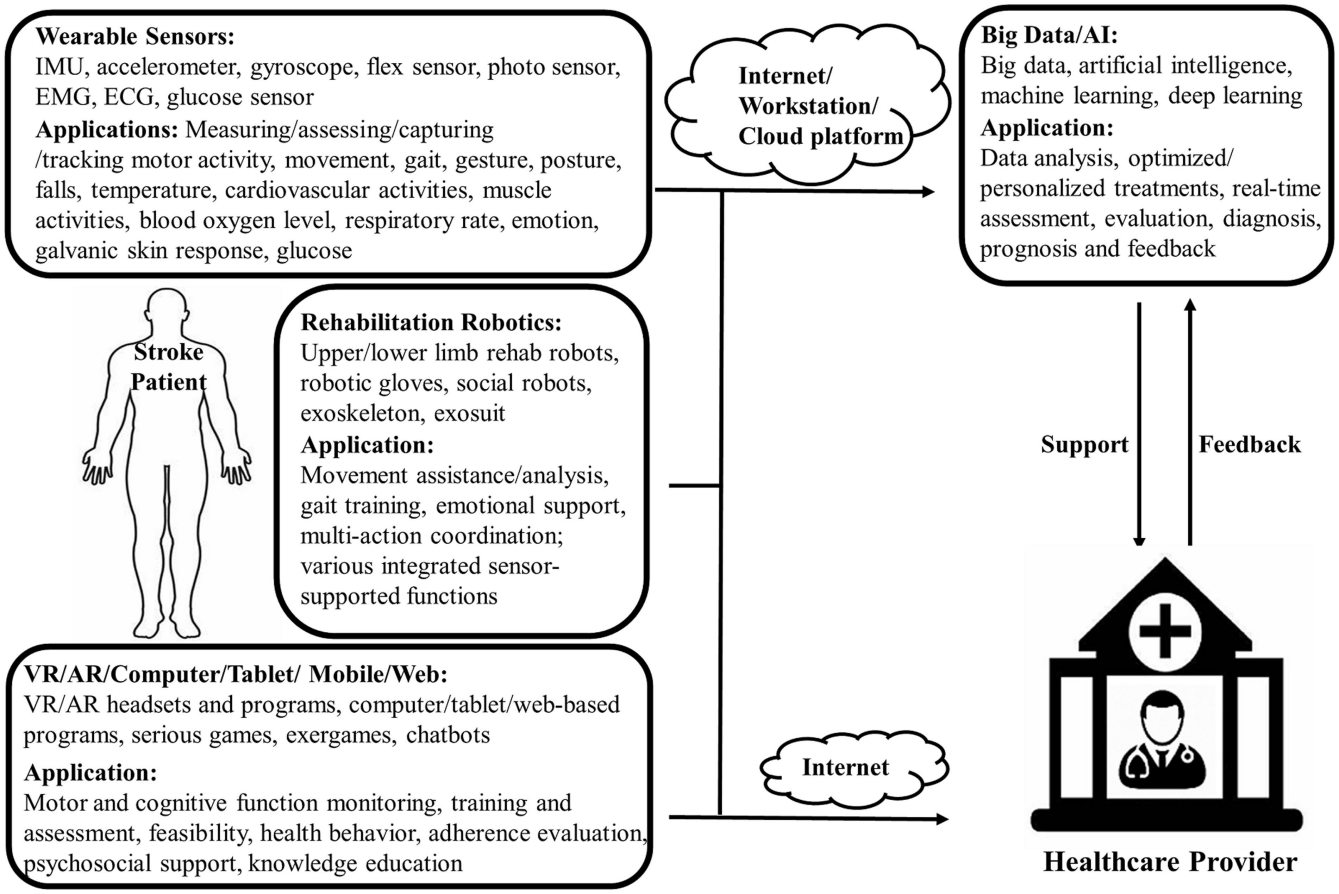
Overview of major technologies in telerehabilitation systems.

Mobile-based, computer-based and web-based ICTs are usually integrated into interventions in the form of games, virtual reality (VR) and other trainings. They can deliver user training data to healthcare professionals for evaluation (17). They also can be combined with videoconferencing for better outcomes. Wearable sensors are used to collect patients’ data in order to monitor their status including falls, heart rate, blood pressure, respiratory rate and blood oxygen levels (24). In a study by Asano et al. (25), 61 patients performed rehabilitation training though tablet-based TR system followed by a review through videoconferencing. Sensers were used to obtain their physiological signals to find adverse effects from TR for safety reason. Nasrabadi et al. (26) developed an activity recognition system based on inertial measurement units (IMU) for TR. The system can be used to track body motion during movement-based therapies in order to detect wrong actions and to assess training effectiveness. Accelerometer, gyroscope and electromyograph (EMG) sensors are also frequently used to track body motion and muscle activities. Furthermore, artificial intelligence (AI) approach was increasingly used in stroke rehabilitation. For example, machine learning methods were adopted as a promising support tool for clinicians to predict functional recovery of stroke patients (27). Major technologies used in TR were summarized in Figure 1.

## 3. Interventions and related technologies in telerehabilitation

According to a highly cited review article, interventions for stroke recovery were divided into four types. They are training interventions, technological interventions, pharmacological interventions and neuromodulation interventions (28). Among them, pharmacological interventions are not directly associated to TR. Thus, we will consider only the other three types of interventions below.

Both training interventions and technological interventions are related to physical activity and exercise. The former is in the form of strength or/and task-oriented trainings, while the latter consists of serious gaming, VR and robotics. These interventions are not mutually exclusive. Instead, they can be combined together to achieve optimal outcomes for stroke recovery. For example, Lee et al. (29) studied the role of a smart glove in upper limb function recovery. Participants were asked to perform task-oriented actions in a VR environment. The control group takes usual care plus recreational activities. As a result, the intervention group demonstrated better outcomes for all measures. Hao et al. (15) reviewed effects of VR-based TR systems including totally 260 stroke patients. The VR-TR group showed similar outcomes to in-person rehabilitation group in terms of upper limb and balance functions. Rozevink et al. (30) studied the effects of an upper limb robot-assisted serious game therapy in a TR setting. Their system significantly improved motor function of patients with high satisfaction and adherence.

Neuromodulation interventions include electrical stimulation and magnetic stimulation for the purpose to enhance neural pathways of different human body systems (24). Electrical stimulation (ES) is a popular and well-established intervention for stroke therapy, and it can be broken down into several subcategories, such as functional electrical stimulation (FES) for peripheral nerve system and transcranial direct current stimulation (tDCS) for central nerve system (31, 32). The effectiveness of ES for stroke recovery has been extensively studied with positive results (33, 34). But these interventions require sufficient knowledge and experience in order to operate ES devices properly. Additionally, there are also safety concerns. As a result, there are very few studies combining ES and TR for stroke recovery. Hermann et al. (35) examined the efficacy of FES treatment for post-stroke arm disorder, while Ko et al. (36) reported the use of home-based tDCS for cognitive training. Their data suggest that using ES in TR is promising. On the hand, transcranial magnetic stimulation (TMS) has been widely used to treat various stroke conditions (37, 38). But it has not been used in TR in our literature search, possibly because TMS device is very expensive and complex. The use of neuromodulation interventions in TR is just starting to receive attention, and further studies are needed to examine their outcomes.

## 4. Effects of telerehabilitation on post-stroke functional impairments

### 4.1. Movement disorders

Majority stroke survivors suffer from movement disorders (39), and rehabilitation practices are the key to help patients to regain their lost abilities (28). In a random clinical trial (RCT), 124 patients with arm motor disorders were equally divided into two groups – TR group and in-clinic therapy group. All patient received thirty six 72-min sessions of identical interventions in the form of daily functional games, exercise videos, and stroke education. The results demonstrated that both groups have significant gains in arm functionality based on Fugl-Meyer (FM) scores with high satisfaction, and there is no significant difference between the two groups. But in-clinic therapy group showed better adherence compared to TR group (40). In a similar study, the authors found that early TR after stroke is suitable for intensive arm motor trainings with excellent feasibility, safety and efficacy (41). Stzurm et al. (42) developed a computer game-assisted TR platform to improve compliance and accessibility of rehabilitation programs for individuals. They found that the TR service resulted significant improvement of patients’ hand-arm functions which were evaluated by Wolf Motor Function Test and a customized computer-based system. Additionally, robotic rehabilitation for motor recovery via TR service substantially improved the upper limb function of patients with high satisfaction (43, 44).

For lower limb related disorders, Held et al. (45) developed an autonomous TR system for balance and gait recovery. During a 12-week period, patients play exercise games in a VR environment for 40 min per session. Their results suggest the TR system is safe, feasible and able to provide intensive therapy at home for lower limb trainings. Lin et al. (46) recruited 24 chronic stroke patients who were asked to perform a 50-min balance training session, and three times each week. The authors found that TR increased balance abilities of patients in terms of Berg Balance Scale, and there is no difference in training effect and satisfaction between TR group and conventional therapy group. In another study, patients reported high acceptability and satisfaction of a serious game-based TR system for ankle movements (47).

TR also plays an important role in the recovery of activities of daily living (ADL) after stroke, which is a key indicator of one’s functional status. In a systematic review, the authors conclude that there is no significant difference between TR intervention group and in-person physical therapy group, as well as usual care group (10). These studies suggest that TR is a feasible and effective way to improve motor functions of stroke patients, and its effects are not inferior to traditional therapies. But augmented TR training may be not effective in improving physical function compared with usual care (48).

### 4.2. Cognitive disorders

Post-stroke cognitive disorders may result in tremendous reduction in quality of life and independence on ADL, and they can also lead to poor adherence to treatments (49). In a study by Faria et al. (50), 36 chronic stroke patients were recruited, and divided into two groups – adaptive VR-based TR group and paper-and-pencil-based control group with task generator. Both groups performed equivalent cognitive trainings for 12 sessions over 1 month. The results showed that the TR group had significant improvement in cognitive functions compared to control group. In another study, VR-based cognitive TR also resulted better outcomes than traditional rehabilitation for stroke patients in terms of global cognitive level, attentive, memory and linguistic skills (51). Additionally, Lawson et al. demonstrated feasibility of TR in cognitive trainings as well as its non-inferiority compared with their previous in-person rehabilitation trainings (52). Bernini et al. (53, 54) also showed TR is not inferior to in-person rehabilitation with satisfiable user experience for general cognitive disorders. Overall, TR system is feasible for cognitive trainings, and has similar or better performance compared to traditional cognitive training methods.

### 4.3. Speech and language disorders

Aphasia has an occurrence rate of 30% in hospitalized stroke patients. It often leads to social isolation and low mood, and was rated as one of the worst diseases that has negative impact on quality of life (55). Meltzer et al. evaluated the effectiveness of TR for communication disorders by conducting identical treatments with 44 patients for TR group and in-person group. After 10-week treatment, all patients had significant improvement on evaluated indices, and the gain is similar for both groups. Their findings suggest that TR is highly effective for communication disorders (56). In another study, Maresca et al. conducted a RCT consisting of 30 patients with aphasia, who were assigned to either control group trained with a conventional treatment or experimental group trained with tablet-based TR platform. After 6-month treatment, the experimental group demonstrated significant improvement in all evaluations expect writing, and performed much better than control group (57). Similarly, a web-based application demonstrated TR is effective way for aphasia training (58). Ora et al. also conducted a RCT consisting of a TR group and a control group with 31 patients for each. Both of them received usual care, but TR group also received additional 5-h training per week. As a result, there is no significant difference between the two groups for assessed indicis after 4 weeks (59). In a another study by the same authors, TR were shown to be a feasible and acceptable way for aphasia training (60). A review suggests intensity of therapy is the key for aphasia trainings (61), thus TR may serve as a complementary intervention for better outcomes.

### 4.4. Other disorders

Approximately 50% of stroke patients have swallowing disorders, and TR composed of motion and muscle exercises can effectively improve swallow functions with high patient satisfaction (62). Wearable EMG sensors can monitor swallowing activities and subsequently detect dysphagia in remote settings (63). TR is also used to reduce post-stroke depression, and telephone intervention demonstrated similar effects in reducing depression to usual care or in-person intervention (64).

## 5. Discussion

As shown in Table 1, TR demonstrated considerable feasibility and effectiveness for stroke recovery. It is not inferior to usual care and in-clinic therapy with matched intensity, duration, and frequency. It also has a high satisfaction rate among stroke patients. But TR is probably not suitable for every patient because of technical barriers and various personal reasons. TR treatments have a higher dropout rate than traditional rehabilitation programs because some patients, especially those with cognitive disorders, have difficulties in completing the training session remotely (50). Additionally, without healthcare professionals standing aside, many patients have less confidence and motivation to conduct interventions, which subsequently results in low adherence and poorer clinical outcomes. Furthermore, some interventions requiring large, expensive or dangerous devices may be not suitable for home settings. There are also some concerns in interpreting TR outcomes. First, inclusion and exclusion criteria for participants are not perfect due to limited availability of patients (40). Second, cohort studies lack control groups, which may lead to wrong conclusions (42). Third, satisfaction and other self-reported data are not subjective (41). On the other hand, a recent survey research regarding telemedicine showed that majority physicians and patients still prefer in-person care, because they do not trust the quality of TR. Lack of physical exam and intervention accuracy were cited as key reasons (65). The results also suggest that self-reported satisfaction rate from patients may be questionable.

**Table 1:** Representative references of telerehabilitation studies.

Study population	Objective	Characteristics of telerehabilitation	Assessment methods	Key findings
124 patients with movement disorders within 4 weeks of stroke (25)	To evaluate the efficacy of a telerehabilitation system	Tablet-based telerehabilitation program including limb strengthening and balance exercises plus videoconference reviews. 60 min per session, 5 times per week over 3 months.	Comparison: usual rehabilitation care.	The intervention and control groups self-reported similar improvements in functional outcomes. No significance was found between them.
			Measures: results were assessed with scores of the late-life function and disability instrument (LLFDI), walking test and modified Barthel index etc.	
124 stroke patients with arm motor disorders within 6 weeks of stroke (40)	To study the efficacy of home-based telerehabilitation vs. in-clinic therapy	Arm motor therapy including exercises and functional trainings plus stroke education through videoconference with the therapist using the computer-based TR system. 70 min per session, totally 36 sessions over 4 weeks.	Comparison: in-clinic therapy with matched intensity, duration, and frequency.	Both TR and in-clinic rehabilitation produced substantial gains in arm motor function. No significance was found between them.
			Measures: results were assessed Fugl-Meyer score, NIHSS score, modified Rankin scale score.	
16 stroke patients with a recent hemiparetic stroke (41)	To evaluate feasibility, safety, and potential efficacy of providing intense TR therapy early after stroke	Functional games, exercise videos, education, and daily assessments via videoconference with the therapist via the computer-based TR system. 70 min per session, totally 18 TR sessions over 6 weeks.	Comparison: 18 in-clinic rehabilitation sessions for the same group of patients with the same intervention program, which are performed alternately with TR.	TR is feasible and safe in stroke recovery at less than 1 month from onset.
			Measures: same as previous one.	
10 single stroke patients with upper limb disorders between 4 months and 2 years (42)	To determine the feasibility and acceptability of a game-assisted home exercise program.	Game-assisted repetitive task practice exercise program consisting of 7 object manipulation tasks. Before TR program, participants received three to four initial clinically supervised therapy sessions. 145–60 min per session, 4 times per week over 6 weeks.	Comparison: same group of patients, before and after interventions.	Feasible trial procedures, acceptable game-assisted task-oriented home training with a high compliance rate and positive outcomes.
			Measures: feasibility and acceptability were based on retention rate, compliance and semi-structured interviews. Quantitative analysis included the Wolf Motor Function and a computerized performance-based assessment.	
14 patients with upper limb disorders at least 6 months after stroke (43)	To assess the effects of robotic home-based treatment rehabilitation	Customized upper limb home-based robotic rehabilitation programs including circle drawing, point-to-point practice, shoulder horizontal abduction, and other exercises.	Comparison: same group of patients, before and after interventions.	Significant improvements in MAS of elbow and computer-based exercise performance
			Measures: NIHSS score, Fugl-Meyer score, Barthel Index, modified Ashworth scale etc.	
15 patients with lower limb disorders 3–74 months after stroke (45)	To study the safety, usability and patient acceptance of an autonomous telerehabilitation system for balance and gait.	Autonomous rehabilitation based on virtual rehabilitation was provided at the participants’ home. 10 to 40 min per day for 12 weeks based on patient conditions.	Comparison: same group of patients, before and after interventions.	The TR system is safe, feasible and can help to intensive rehabilitative therapy at home.
			Measures: compliance and acceptance of the system measured with the technology acceptance model (TAM).	
32 patients with movement disorders at least 6 months after stroke (46)	To examine the possible effects of therapeutic exercises performed by an App on trunk control, balance, and gait in stroke survivors.	Videoconference with the therapist via the computer-based TR system with wireless sensors to monitor patient conditions. 10 min of standing exercise and 10 min of 3D interactive games, 50 min per session per day for 4 weeks.	Comparison: usual care.	No significant difference between groups could be demonstrated. Some unwillingness to use TR system.
			Measures: assessed with berg balance scale (BBS), Barthel index (BI), and self-reported telerehabilitation satisfaction of the participants	
95 first-ever stroke patients within 2 weeks of discharge (48)	To investigate whether augmented TR intervention improved physical function compared with usual care	Telephone and text-based TR services with personalized treatment plans consisting of 5 sessions per week for 6 months.	Comparison: usual care.	Augmented TR Intervention may be effective in preventing deterioration but no significant difference from usual care in improving physical function.
			Measures: Stroke Impact Scale (SIS3.0), hand grip strength, balance test etc.	
36 patients with cognitive disorders at least 6 months after stroke (50)	To test effectiveness of VR rehabilitation intervention for cognitive disorders	Customized application Reh@City v2.0 providing adaptive cognitive training experience through everyday tasks VR simulation. 90 min per session for 2 months	Comparison: content-equivalent paper-and-pencil training.	TR showed higher effectiveness with improvements in different cognitive domains and self-perceived cognitive deficits in everyday life, but with higher dropout rate.
			Measures: general cognitive functioning; attention; WMS-III; self-reported evaluation.	
40 patients with cognitive disorders, 3–6 months after stroke (51)	To evaluate the efficacy of a VR-based TR system	VR-based cognitive training system with home tablet. 50 min per session, 3 sessions per weeks for 6 months	Comparison: standard cognitive training.	TR has significant improvement in global cognitive level, as well as in the attentive, memory and linguistic skills.
			Measures: Montreal overall cognitive assessment, attentive matrices, phonemic fluency etc.	
46 patients with cognitive disorders at least 3 months after stroke (52)	To determine usability of a TR-based cognitive training system.	Zoom-based TR via videoconference plus traditional in-person rehabilitation. 120 min per week for 6 weeks for TR.	Comparison: before and after interventions, and previous patients in similar conditions.	TR is a feasibility an option for remote delivery of compensatory memory skills training after a stroke.
			Measures: Goal Attainment Scaling (GAS), Comprehensive Assessment of Prospective memory (CAPM), adherence, self-reported questionnaires.	
40 patients with mild or major cognitive disorders (53)	To compare the same rehabilitation program performed at home and at hospital	Computer-based intervention based on traditional paper-and-pencil exercises at home via videoconference. 45 min per session, 3 sessions per week for 6 weeks.	Comparison: in-person cognitive intervention with the same computer-based exercises.	Effects of TR are not inferior to in-clinic rehabilitation. No significance between two groups.
			Measures: exhaustive neuropsychological battery before and after the intervention.	
46 patients with cognitive disorders at least 3 months after stroke	To determine usability and user experience of a TR-based cognitive training system.	Zoom-based TR via videoconference plus traditional in-person rehabilitation. 120 min per week for 6 weeks for TR.	Comparison: before and after interventions, and previous patients in similar conditions.	The study supports the feasibility and potential effectiveness of TR options for remote delivery of compensatory memory skills training after a stroke
			Measures: Goal Attainment Scaling (GAS), Comprehensive Assessment of Prospective memory (CAPM), adherence, self-reported questionnaires.	
40 patients with cognitive disorders, 3–6 months after stroke (51)	To evaluate the efficacy of a VR-based TR system	VR-based cognitive training system with home tablet. 50 min per session, 3 sessions per weeks for 6 months	Comparison: standard cognitive training.	TR has significant improvement in global cognitive level, as well as in the attentive, memory and linguistic skills.
			Measures: Montreal overall cognitive assessment, attentive matrices, phonemic fluency etc.	
44 patients with communication disorders at least 6 months after stroke (56)	To evaluate the effectiveness of TR for communication disorders.	Tablet-based home exercises and realistic, customized treatment plans tailored to the needs of each individual client. Weekly 60-min sessions for 10 weeks	Comparison: in-person rehabilitation.	No significant difference of all except self-rated confidence having higher score for in-person group.
			Measures: western aphasia battery aphasia quotient, cognitive-linguistic quick test, communication effectiveness index, confidence ratings etc.	
30 patients with aphasia due to stroke (57)	To evaluate the effectiveness of a TR training for aphasia using a VR system.	Virtual reality rehabilitation system. 50 min per session, 5 sessions per week for 6 months. and included 2 phases	Comparison: traditional linguistic treatment using paper-pencil tools.	The experimental group improves in all the investigated areas, except for writing, while the control group only improves in comprehension, depression, and quality of life
			Measures: neuropsychological evaluation including token test, aphasic depression rating scale etc. before and after intervention.	
32 patients with aphasia at least 6 months after stroke (58)	To investigate an intensive asynchronous computer-based treatment delivered remotely with clinician oversight to people with aphasia.	A web-based TR application – Web ORLA® (Oral Reading for Language in Aphasia) which provides repeated choral and independent reading aloud of sentences with a virtual therapist. 90 min per day, 6 days per week for 6 weeks	Comparison: commercially available computer games.	Improved language outcomes following intensive, web-based delivery of ORLA® to individuals with chronic aphasia.
			Measures: Western Aphasia Battery; Communicative Abilities in Daily Living Test.	
62 post-stroke patients with aphasia (59)	To study effects of augmented rehabilitation via TR for speech-language disorders	TR via videoconference in addition to usual care. 5 h per week for 4 weeks.	Comparison: usual care alone.	TR via videoconference may be a viable rehabilitation model. But additional TR training has no additional gains.
			Measures: Norwegian Basic Aphasia Assessment, Verb and Sentence Test score.	

To address above-mentioned issues, technological advances are the key. Intelligent devices requiring less efforts from patients can overcome technical barriers in usability. VR and haptic devices can be used to create an environment mimicking clinic setting to increase confidence and motivation of patients (66, 67). Additionally, many devices for stroke interventions can be redesigned to adapt TR platforms. For example, FES and tDCS devices have already been used for neuromodulation in TR (68), but the number of studies is very limited mainly for safety reasons. With additions of remote control and extensive safety mechanisms, ES devices have potential to be used in TR more frequently. To address the lack of physical exam and accuracy in TR, wearable sensors can be used to acquire a variety of parameters of patients and to monitor their health conditions and activities (69, 70). Thus, healthcare professionals can detect adverse effects during TR and make better intervention plans.

With further development of technologies, fully digitalized TR system may be possible. TR-based interventions, which combine serious games, immersive VR, rehabilitation robots and various sensors, have possibility to achieve better outcomes. Patients’ data can be collected with sensors and analyzed though machine learning approach. Traditional measures for evaluating intervention outcomes, such as Berg balance scale, can be performed automatically with proper devices (71, 72). Besides technology aspects, new TR models should also be considered. Community health workers and caregivers have received much attention (73, 74), but their roles in TR were not fully explored. They can serve as a bridge between healthcare professionals and patients to overcome certain communication-related issues and technical barriers. Overall speaking, TR is still in its developing stage, and further studies are needed to provide evidence for optimal use of TR.

## Author contributions

YX and QW wrote and finalized the manuscript. JX and BZ provided support in reference searching. All authors contributed to the article and approved the submitted version.

## Funding

The research was supported by Research Startup Funding of Shantou University #NTF21014 to YX.

## Conflict of interest

The authors declare that the research was conducted in the absence of any commercial or financial relationships that could be construed as a potential conflict of interest.

## Publisher’s note

All claims expressed in this article are solely those of the authors and do not necessarily represent those of their affiliated organizations, or those of the publisher, the editors and the reviewers. Any product that may be evaluated in this article, or claim that may be made by its manufacturer, is not guaranteed or endorsed by the publisher.
