# Supplementary material for: ICTs and interventions in telerehabilitation and their effects on stroke recovery
Source: Front Neurol. 2023 Aug 14;14:1234003. doi: 10.3389/fneur.2023.1234003 (PMC10460969; doi:10.3389/fneur.2023.1234003)
Supplement: Supplementary file 1 [file Data_Sheet_1.PDF]

Reference selection criteria:

We searched two databases – web of science and PubMed. The keywords used are “telerehabilitation’ and “stroke”. We are mainly focusing on clinical trials within recent 5 years.

But for some topics, for example telerehabilitation of stroke with FES, there is no article available, we used a clinical trial more than 5 years for reference. We also used some classic and highly-cited reviews in introduction part.

There are about 50 clinical trials in recent 5 years using keywords: stroke and telerehabilitation and most of them are for movement disorders. Since this is not a systematic review, we did not cite all of them. We only chose those fitting for our topics. Some trials have no clear results were also ignored. Additionally, for those articles with similar conclusions, we only selected few representative articles.
